# Evaluation of a new protocol for rapid identification of *Streptococcus pneumoniae* in blood cultures using the modified bile solubility test: Gram staining is still standing

**DOI:** 10.1128/jcm.01222-24

**Published:** 2024-12-18

**Authors:** Antoine Aupaix, Alexia Verroken, Hector Rodriguez-Villalobos

**Affiliations:** 1 Department of Microbiology, Cliniques Universitaires Saint-Luc, Catholic University of Louvain 81803 , Brussels, Belgium; NorthShore University HealthSystem Department of Pathology and Laboratory Medicine, Evanston, Illinois, USA

**Keywords:** *Streptococcus pneumoniae*, blood culture, MALDI-TOF, bile solubility, optochin, rapid identification

## Abstract

This study aimed to evaluate a new protocol of the bile solubility test performed directly on the blood from positive blood culture bottles to identify *Streptococcus pneumoniae* rapidly. Seventy-five positive blood cultures (PBC) showing Gram-positive cocci in pairs or chains on Gram stain, including 32 *S. pneumoniae* isolates and three reference American Type Culture Collection (ATCC) isolates were included to evaluate the performance of a modified bile solubility test (MBST). One milliliter of blood from the PBC bottle was mixed with 0.5 mL of 10% desoxycholate or a saline solution. Both suspensions were analyzed after 10 min of incubation through a Gram stain to detect solubilization. This technique was compared with matrix-assisted laser desorption/ionization time-of-flight mass spectrometry identification, performed on PBC following extraction or on colonies after short or standard incubation, and the optochin susceptibility test on colonies. The capsular serotypes were determined for all *S. pneumoniae*, and the Belgian National Reference Center confirmed the identification. All 32 clinical isolates and the ATCC isolate of *S. pneumoniae* were solubilized on the desoxycholate-treated slides, while the other species tested remained visually unchanged on both, the test and control slides. The MBST test demonstrated a 100% sensitivity and specificity with a mean turnaround time (TAT) of just 39 min, making it 14 h and 56 min faster than the optochin susceptibility test. This rapid variant of the bile solubility test appears to be a reliable method to identify *S. pneumoniae* directly from positive blood culture bottles, with a TAT of 39 min. It is a cost-effective, easy-to-perform, and time-efficient technique. Negative results should be interpreted cautiously, as they may result from mixed infections with *S. pneumoniae* and other Gram-positive cocci.

## INTRODUCTION

*Streptococcus pneumoniae*, a Gram-positive bacterium and commensal from mucosal surfaces of upper airways, remains one of the most deadly pathogens in humans (1). It can cause a wide range of diseases, including bloodstream infections (BSI), pneumonia, meningitis, otitis, and arthritis. BSI with *S. pneumoniae* have a high mortality rate, and the capsular serotype plays a major role in the pathogenesis, partially by resisting the complement C3 (2). *Streptococcus pneumoniae* blood stream infections represent a high mortality risk (3), and their rapid identification is warranted for appropriate antimicrobial therapy and better clinical outcomes (4). The development of *matrix-assisted laser desorption/ionization* time-of-flight mass spectrometry (MALDI-TOF MS) has allowed reliable and rapid identification of bacteria and yeast from young subcultures after a few hours of incubation and even directly from positive blood culture (PBC) bottles, with a turnaround time (TAT) of 10 to 40 min from the time of positivity (5, 6). However, it is still a limited tool to distinguish *S. pneumoniae* from other closely related species of the *Streptococcus mitis* group (7, 8). This distinction is important as the former is usually considered highly pathogenic, and the latter is less frequently associated with true clinical infection. Moreover, rapid identification allows early antimicrobial therapy and can improve clinical outcomes.

This limitation also extends to direct identification from positive blood cultures by MALDI-TOF MS, where mismatches are predominantly observed in Gram-positive bacteria (7). Among those, *S. pneumoniae* appears particularly challenging to identify accurately, with identification rates ranging from 15% to 71.5% based on the direct identification protocol used (6, 9). Even with molecular techniques, such as 16S RNA sequencing, it is challenging to differentiate effectively between *S. oralis*, *S. mitis*, and *S. pneumoniae* due to their DNA sequence similarities (10).

Although real-time PCR targeting different genes (i.e., lytA, piaB) is usually considered the gold standard method for the identification of *S. pneumoniae*, the following two conventional methods are still considered as a reference in clinical laboratory practice: the optochin susceptibility test and bile solubility test (11). The first assay does not have one standardized procedure, and the performance of the test varies according to the culture, media, and reading time points (12). A growing limitation of the optochin susceptibility test is the high rate of optochin resistance in *S. pneumoniae* isolates, which can be as high as 18%, reducing the assay’s sensitivity (11). Some strains can also give false-positive results, notably *S. pseudopneumoniae*, if incubated in ambient air. The bile solubility test also exists in different variants but is less commonly used due to subjectivity in the interpretation and lack of reproducibility (13). Some strains of *S. pneumoniae* may be non-soluble in bile (notably non-capsulated strains), and bile-soluble strains of *S. mitis* have been described. Bile solubility generally performs better than optochin susceptibility (11, 14). Both assays lack standardization, requiring full-grown colonies before use, enabling an identification result up to 1 day after PBC detection.

This study evaluated an accelerated protocol called the modified bile solubility test (MBST) as an alternative to the bile solubility test. First described in 1979 (15) and then, to our knowledge, no longer studied in the literature since 1983, this technique allows the identification of *S. pneumoniae* directly on the blood of a PBC bottle using microscopy.

## MATERIALS AND METHODS

### Isolates selected

The study was conducted at the microbiology laboratory of the Cliniques Universitaires Saint-Luc (a 960-bed tertiary hospital in Brussels, Belgium). We included 75 patient blood culture bottles (BD BACTEC Plus Aerobic/F and Lytic/10 Anaerobic/F Culture Vials) detected positive by the BD BACTEC FX incubators (Becton Dickinson, Franklin Lakes, New Jersey, USA) and showing Gram-positive cocci in chains or pairs. Only one sample was included per patient. The microscopic examination and identification time were recorded to assess any time saved toward *S. pneumoniae* identification. Three reference strains (American Type Culture Collection [ATCC] 49,619 *S*. *pneumoniae*, ATCC 12,836 *S*. *agalactiae*, ATCC 29212 *E. faecalis*) were included as positive and negative controls, respectively.

### Routine workflow (standard method)

Blood culture bottles detected positive were identified by MALDI-TOF MS with the Microflex LT (Bruker, Bremen, Germany) using MBT Compass IVD 4.2 (Version [90]) and MBT IVD Library DB8326 MSP (MaldiBiotyperDBUpdate_V9 or V12). According to the algorithm developed by Verroken et al. (16), identification was either performed on colonies after standard incubation for PBC detected between 3 and 10 p.m. the previous day, on 3- to 5-h subcultured colonies for PBC detected before 11 a.m. on the same day, or directly on the blood of PBC bottles following protein extraction with the Sepsityper kit (Bruker, Bremen, Germany) for PBC retrieved between 11 a.m. and 3 p.m. For identification on colonies and direct identification with Sepsityper kit, cut-off scores to accept the results were respectively 2.00 and 1.80, as recommended by the manufacturer. This library matching score is measured by comparing the number, intensity, and symmetry of peaks in the spectrum obtained with reference spectra of known species registered in the MALDI-TOF library. The score ranges from 0 to 3, with higher match scores indicating more accurate identification. Additionally, results were not accepted if more than one species was identified above the cut-off score, indicating either a mixed culture or the inability of MALDI-TOF MS to discriminate between those species. When microscopic examination on blood was positive for Gram-positive cocci in chains, an optochin susceptibility test was performed by placing an optochin disk (Optochin, Bio-Rad, Marnes-la-Coquette, France) onto the inoculated surface of a 5% blood sheep agar (Columbia CAP Selective Agar with Sheep Blood, Thermo Fisher Scientific, Massachusetts, USA). Optochin susceptibility is used to differentiate the susceptible species *S. pneumoniae* from other alpha-hemolytic streptococci that are resistant to this molecule. After 16–18 h of incubation, a diameter of the inhibition zone >13 mm was interpreted as positive. For all *S. pneumoniae* isolates, identification was confirmed in the Belgian National Reference Center (Universitair Ziekenhuis Leuven, Belgium). They use a combination of three tests: optochin susceptibility, bile solubility, and serotyping performed by phase-contrast microscopy using the Quellung reaction with pool, group, and serotype sera (SSI Diagnostica, Hillerød, Denmark). For difficult-to-identify strains, lytA PCR and whole-genome sequencing are used.

### Modified bile solubility test

**Fig 1:**
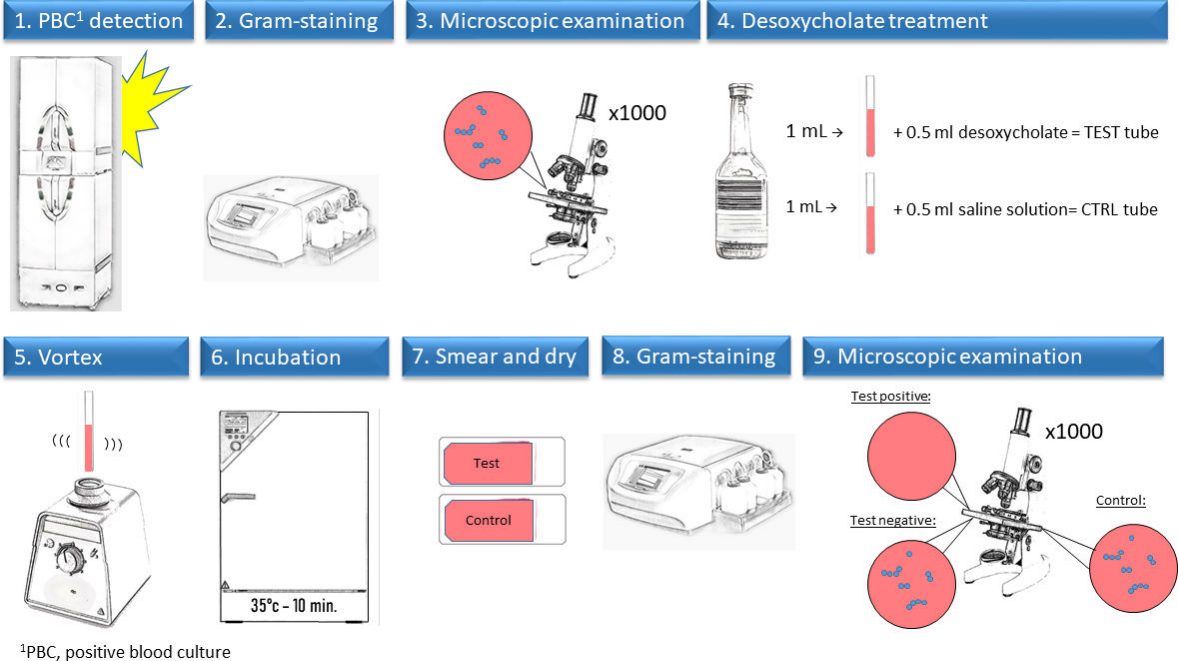
Schematic representation of the workflow of the modified bile solubility test.

Bile solubility tests are based on the specific autolysis of pneumococcal cells in the presence of bile salts. Unlike standard tests, this MBST is assessed microscopically. A schematic representation of the workflow of the MBST is shown in Fig. 1. Briefly, 1 mL of blood from a PBC bottle showing a Gram stain with Gram-positive cocci in chains or pairs was mixed with 0.5 mL of 10% sodium desoxycholate solution (BBL Desoxycholate Reagent Droppers, Becton Dickinson and Company, Maryland, USA) in a test tube and with 0.5 mL of saline solution in a “control tube.” Both tubes were then incubated at 35°C for 10 min. A single drop of the suspensions was then smeared on slides, dried at room temperature, and Gram stained to detect solubilization. This protocol is a shorter version of a protocol published in a previous study in 1983 (17).

## RESULTS

A total of 78 isolates were tested, including three ATCC reference isolates, 32 clinical isolates of *S. pneumoniae* (confirmed and serotyped by the reference center), and 43 other clinical isolates described as cocci in chains or pairs with Gram stain (Table 1).

**TABLE 1:** Detailed capsular serotypes of *S. pneumoniae* and identification of other species included

*Streptococcus pneumoniae* capsular serotype	N	Other cocci in chains or in pairs	*N*	ATCC isolates
Serotype 3	4	*Enterococcus faecium*	10	ATCC 49619 *Streptococcus pneumoniae*
Serotype 6C	3	*Enterococcus faecalis*	5	ATCC 29212 *Enterococcus faecalis*
Serotype 8	5	*Enterococcus gallinarum*	1	ATCC 12836 *Streptococcus agalactiae*
Serotype 9N	2	*Streptococcus pyogenes*	2	
Serotype 10A	2	*Streptococcus agalactiae*	6	
Serotype 11A	3	*Streptococcus dysgalactiae*	2	
Serotype 12F	4	Former *Streptococcus bovis* group	3	
Serotype 15B	1	*Streptococcus anginosus* group	5	
Serotype 15C	1	*Streptococcus mitis* group	7	
Serotype 17F	1	*Streptococcus salivarius* group	1	
Serotype 19A	2	*Leuconostoc lactis*	1	
Serotype 22F	1			
Serotype 35F	3			
**Total**	32		43	

**Fig 2:**
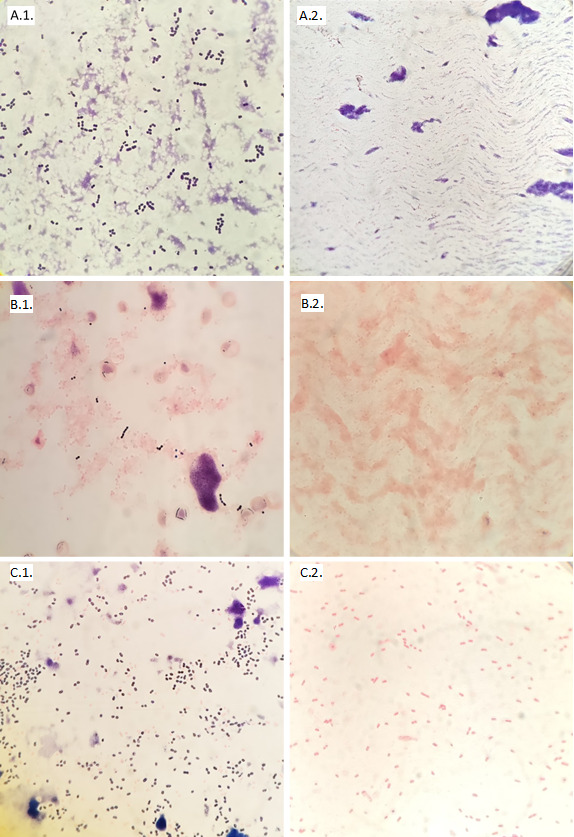
Gram stain (×1,000 magnification) of ATCC 49,619 *S*. *pneumoniae* with physiological serum (A.1.) and with desoxycholate (A.2.); *S. pneumoniae* from a clinical sample with physiological serum (B.1.) and with desoxycholate (B.2.); *S. pneumoniae* and *E. coli* from a clinical sample with physiological serum (C.1.) and with desoxycholate (C.2.).

**Fig 3:**
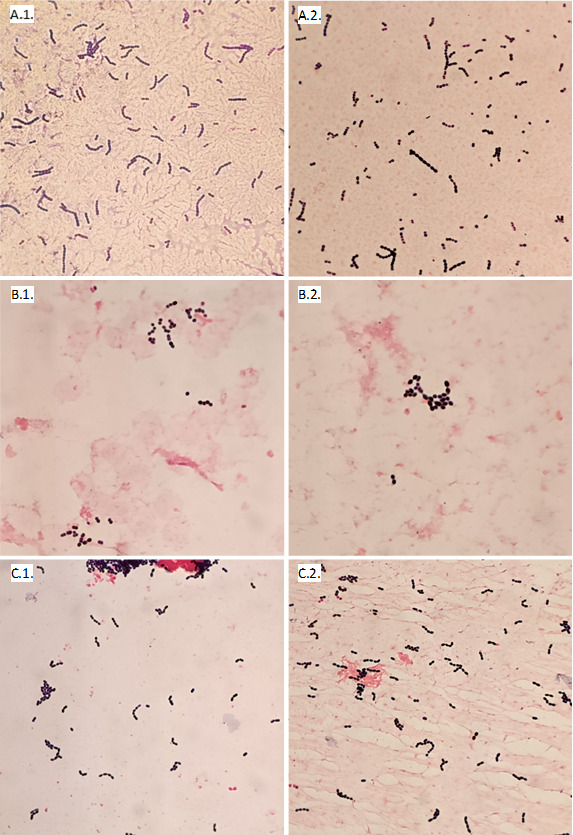
Gram stain (×1,000 magnification) of *S. pyogenes* from a clinical sample with physiological serum (A.1.) and with desoxycholate (A.2.); *E. faecium* from a clinical sample with physiological serum (B.1.) and with desoxycholate (B.2.); *S. mitis* group from a clinical sample with physiological serum (C.1.) and with desoxycholate (C.2.).

All *S. pneumoniae* isolates were lysed on the smear treated with desoxycholate, giving a sensitivity of 100%. All other species remained unaltered after desoxycholate treatment, giving a specificity of 100%(Table 2). Microscope photographs of the main species tested from the test tubes and controls are shown in Fig. 2 and 3. The MBST and optochin susceptibility showed a positive percentage agreement (PPA) of 100% with the reference method. For MALDI-TOF MS with the standard method, carried out on 18- to 24-h colonies, PPA was 63% (12 isolates with a score above the cut-off out of 19 strains tested). MALDI-TOF MS yielded *S. pneumoniae* identification in four other isolates; however, other species were also reported with scores above 2.0 (*S. mitis*, *S. oralis* and *S. pseudopneumoniae*), making it impossible to discriminate between them. The Sepsityper method was carried out on three isolates, but no identification was achieved as all scores were under 1.80. Notably, the PPA for the Sepsityper method in this study was 0%. MALDI-TOF MS on young colonies was not performed, as none of the included *S. pneumoniae* isolates showed sufficient growth after 3–5 h of incubation.

**TABLE 2:** Results of MBST, optochin susceptibility, and MALDI-TOF MS for *S. pneumoniae* isolates and percent positive agreement with reference method^*a*^

Isolate no.	Optochin susceptibility	Modified bile solubility test	MALDI-TOF MS: all different isolate identifications from one sample spot and score^*b*^			
			Method^*c*^	1st identification	2nd identification	3rd identification
1	Susceptible	Positive	Sepsityper	*S. pneumoniae* (1.68)	*S. mitis* (1.67)	*S. oralis* (1.61)
2	Susceptible	Positive	Sepsityper	*S. pneumoniae* (1.77)	*S. mitis* (1.67)	*S. oralis* (1.53)
3	Susceptible	Positive	Sepsityper	Failed: no peak found		
4	Susceptible	Positive	Standard	*S. pneumoniae* (1.87)	*S. oralis* (1.77)	*S. pseudopneumoniae* (1.52)
5	Susceptible	Positive	Standard	*S. pneumoniae (1.77*)	*S. mitis/oralis (1.71*)	
6	Susceptible	Positive	Standard	*S. pneumoniae (1.97*)	*S. mitis/oralis (1.93*)	
7	Susceptible	Positive	Standard	*S. pneumoniae* (2.13)	*S. mitis* (2.05)	*S. oralis* (2.02)
8	Susceptible	Positive	Standard	*S. pneumoniae* (2.48)	*S. pseudopneumoniae* (2.44)	
9	Susceptible	Positive	Standard	*S. pneumoniae* (2.38)	*S. pseudopneumoniae* (2.29)	
10	Susceptible	Positive	Standard	*S. pneumoniae* (2.34)	*S. pseudopneumoniae* (2.23)	
11	Susceptible	Positive	Standard	*S. pneumoniae* (2.07)		
12	Susceptible	Positive	Standard	*S. pneumoniae* (2.31)		
13	Susceptible	Positive	Standard	*S. pneumoniae* (2.34)		
14	Susceptible	Positive	Standard	*S. pneumoniae* (2.21)		
15	Susceptible	Positive	Standard	*S. pneumoniae* (2.34)		
16	Susceptible	Positive	Standard	*S. pneumoniae* (2.05)		
17	Susceptible	Positive	Standard	*S. pneumoniae* (2.27)		
18	Susceptible	Positive	Standard	*S. pneumoniae* (2.17)		
19	Susceptible	Positive	Standard	*S. pneumoniae* (2.02)		
20	Susceptible	Positive	Standard	*S. pneumoniae* (2.28)		
21	Susceptible	Positive	Standard	*S. pneumoniae* (2.44)		
22	Susceptible	Positive	Standard	*S. pneumoniae* (2.35)		
23	Susceptible	Positive	Not performed			
24	Susceptible	Positive	Not performed			
25	Susceptible	Positive	Not performed			
26	Susceptible	Positive	Not performed			
27	Susceptible	Positive	Not performed			
28	Susceptible	Positive	Not performed			
29	Susceptible	Positive	Not performed			
30	Susceptible	Positive	Not performed			
31	Susceptible	Positive	Not performed			
32	Susceptible	Positive	Not performed			
PPA^*d*^	32/32 = 100%	32/32 = 100%	Sepsityper : 0/3 = 0% Standard method : 12/19 = 63%			

^*a*^ Phase-contrast microscopy using the Quellung reaction with serotype-/serogroup-specific sera (SSI Diagnostica, Hillerød, Denmark) together with optochin susceptibility and bile solubility,. For difficult-to-identify strains, lytA PCR and whole-genome sequencing are used.

^*b*^ Only the different identifications are reported. For isolate Nos. 8. to 10. The eight other identifications were *S. pneumoniae*; for isolates 11. to 22, all 10 results were *S. pneumonia*e.

^*c*^ Sepsityper consists of MALDI-TOF MS identification performed directly on blood from a PBC bottle after protein extraction. The standard method reflects the use of MALDI-TOF MS on colonies after 18–24 h of incubation. Rapid identification on young 3- to 5-h colonies was not performed for *S. pneumoniae* isolates in this study. Isolates 1 to 4 and 7 to 18 were identified with MaldiBiotyperDBUpdate_V9, isolates 5, 6 and 19 to 22 with MaldiBiotyperDBUpdate_V12.

^*d*^ PPA, positive percent agreement.

From PBC detection, the mean turnaround time of the MBST was 39 min (TAT range from 36 to 45 min). This assay more rapidly identified *S. pneumoniae* in 100% (32/32) of cases compared to optochin susceptibility, with a mean delta of 896 min or 14 h and 56 min (interquartile range: 4.6–22.0 h).

## DISCUSSION

The modified bile solubility test is a rapid and microscopically assessed variant of the standard, colony-based bile solubility tests. The first method that used bile solubility testing and Gram staining directly on a positive blood culture was published in 1979 (15): in this study, for positive blood cultures (performed in both Thiol broth and tryptic soy broth bottles) showing streptococci on a Gram-stained smear, a second slide was prepared with one drop of blood mixed with one drop of 2% sodium desoxycholate directly smeared on one half of the slide, and one drop of blood mixed with one drop of water on the other half. In contrast to our study, there was no incubation after desoxycholate treatment before slide preparation. In the 1979 study, out of 54 isolates tested, 20 *S*. *pneumoniae* were fully solubilized with desoxycholate, while only one streptococcus group D isolate (identified using biochemical tests: growth in 40% bile and esculin hydrolysis) was completely solubilized among the 34 other streptococci tested. This false-positive reaction was encountered with a 5-day culture in Thiol broth. The isolate was not solubilized with sodium desoxycholate when retested from freshly inoculated broths. As recommended for the standard bile solubility tests, this modified bile solubility test should be performed on fresh colony culture to avoid false-negative or -positive results (18).

In 1983, a modified protocol of this method involved testing simulated blood cultures (17). The authors used 1 mL of blood sampled from positive BACTEC bottles with 0.5 mL of a solution of 10% desoxycholate and 0.5 mL of sterile distilled water in separate tubes, followed by a 30-min incubation at 37°C. From the 14 *S*. *pneumoniae* isolates and 32 other streptococci tested, only one false-negative result was caused by a mixed culture of *S. pneumoniae* and *Staphylococcus epidermidis*.

Our study is the first to validate the MBST on modern blood culture formulations. We confirm the good performance found in previous papers cited above. Completing in 39 min, the turnaround time is equivalent to direct MALDI-TOF MS, demonstrating comparable performance with the optochin susceptibility test.

However, caution should be taken in case of negative results. The persistence of Gram-positive cocci after desoxycholate treatment does not definitively rule out S. *pneumoniae*. A mixed bloodstream infection involving *S. pneumoniae* and other *Streptococcus* species could results in a negative result.

Compared to optochin susceptibility testing, MBST saves several hours in identifying *S. pneumoniae*, potentially impacting the management of bloodstream infections involving this bacterium. Prospective clinical studies would be required to validate this potential clinical impact. Compared with MALDI-TOF MS, the MBST performs better. These findings are supported by a broad literature regarding the limitations of mass spectrometry in discriminating *S. pneumoniae* from close species among the *S. mitis* group.

Recent papers have suggested that MALDI-TOF MS, with peak analysis and updated databases, can correctly discriminate between those species, but further studies are needed to confirm those findings (19).

In the modern world of clinical microbiology, molecular methods are becoming increasingly important, including in the diagnosis of sepsis. Several multiplex PCR systems performed on blood or PBC have been developed to allow rapid identification of bacteria causing BSI. Many authors have investigated different kits, but with relatively few *S. pneumoniae* and even fewer *S. mitis* strains included. One study noted a low sensitivity of the Septifast kit on Lightcycler 2.0 instrument (Roche Diagnostics, GmbH, Mannheim, Germany) explained by a high cut-off precisely set to avoid cross-reaction with alpha-hemolytic streptococci (20). Several studies showed excellent performance of the Biofire Blood Culture Identification Panel 2 (BioFire BCID2, BioFire Diagnostics, Marcy-L’étoile, France) testing *S. pneumoniae* (21–24) as well as with ePlex Blood Culture Identification Panels (ePlex BCID-GP, Roche Diagnostics, GmbH, Mannheim, Germany) (25) and Molecular Mouse Sepsis Panel (Alifax, Polverara, Italy) (26). Sensitivity and specificity of these methods were of 100% but with a low number of *S. pneumoniae* and *S. mitis* group included. False-positive results have been reported with the Verigene Blood Culture–Gram-Positive kit (Verigene BC-GP, DiaSorin, S.p.A., Saluggia, Italia). In this study (27), seven clinical strains of *S. mitis* group were misidentified as *S. pneumoniae* (out of 205 strains of streptoccoci other than *S. pneumoniae*). These molecular assays are certainly of interest, with TATs as short as 1 h. They can detect specific resistance genes in addition to isolate identification, enabling rapid adaptation of empirical antibiotic therapy. In Belgium, these methods are still not widely used in routine practice, given their high cost compared with conventional techniques, and the fact that molecular biology tests are not reimbursed by the national health and disability insurance for this indication.

The first limitation of this study is that we did not test all capsular serotypes nor non-capsulated *S. pneumonia*e. However, the most frequent serotypes associated with invasive infections in Belgium, i.e., serotypes 8, 3, 22F, 19A, 12F, have all been tested. Only serotype 4, responsible for 9.6% of invasive infections in 2023, has not been tested. The 13 serotypes included in this study account for 64% of invasive infections with *S. pneumoniae* in Belgium (data from 2023) (28). Another limitation is that this study was not double blind. Technologists were asked to read these slides as routine Gram tests for blood cultures. Thus, in the absence of easily visualized bacteria, they examine the entire slide before giving a negative result. However, it is possible that they were aware of the MALDI-TOF results when they performed the MBST, which could have biased the test assessments. Finally, we only included seven strains of *S. mitis* group, which represents the main source of misidentification. A panel including more significant number of these species would be useful to validate our method more thoroughly.

In conclusion, this MBST appears to be a reliable test to quickly identify *S. pneumoniae* directly from positive blood cultures. It is easy to perform, requires minimal hands-on-time, and is and is cost effective making it a valuable addition to laboratories in both high- and low-income settings. Negative results should be interpreted cautiously, as they may result from mixed infections with *S. pneumoniae* and other Gram-positive cocci.
